# Roles of peripheral immune cells in the recovery of neurological function after ischemic stroke

**DOI:** 10.3389/fncel.2022.1013905

**Published:** 2022-10-21

**Authors:** Zhaolong Zhang, Mengfei Lv, Xin Zhou, Yu Cui

**Affiliations:** ^1^Department of Interventional Radiology, The Affiliated Hospital of Qingdao University, Qingdao, Shandong, China; ^2^Institute of Neuroregeneration and Neurorehabilitation, Qingdao University, Qingdao, Shandong, China; ^3^Qingdao Medical College, Qingdao University, Qingdao, Shandong, China

**Keywords:** ischemic stroke, functional recovery, T cells, monocytes and macrophages, B cells, neutrophils, cytokines

## Abstract

Stroke is a leading cause of mortality and long-term disability worldwide, with limited spontaneous repair processes occurring after injury. Immune cells are involved in multiple aspects of ischemic stroke, from early damage processes to late recovery-related events. Compared with the substantial advances that have been made in elucidating how immune cells modulate acute ischemic injury, the understanding of the impact of the immune system on functional recovery is limited. In this review, we summarized the mechanisms of brain repair after ischemic stroke from both the neuronal and non-neuronal perspectives, and we review advances in understanding of the effects on functional recovery after ischemic stroke mediated by infiltrated peripheral innate and adaptive immune cells, immune cell-released cytokines and cell-cell interactions. We also highlight studies that advance our understanding of the mechanisms underlying functional recovery mediated by peripheral immune cells after ischemia. Insights into these processes will shed light on the double-edged role of infiltrated peripheral immune cells in functional recovery after ischemic stroke and provide clues for new therapies for improving neurological function.

## Introduction

Stroke is the leading cause of adult disability, and approximately sixty percent of survivors have motor, sensory, memory or language function deficits (Carmichael, 2006). There are two major types of strokes: ischemic stroke and intracerebral hemorrhage. Ischemic stroke occurs when a vessel supplying blood to the brain is obstructed, and accounts for 70–80% of all stroke cases (Feigin et al., 2009). Ischemic stroke can initiate neuronal death, which further result in blood–brain barrier (BBB) disruption and neuroinflammation cascades, leading to severe neurological deficits (Iadecola and Anrather, 2011; Lyu et al., 2021). Generally, the adult brain is considered to have an extremely limited capacity for regeneration. Nevertheless, a growing body of literature suggests that diseases or injuries can trigger molecular, vascular, glial, neuronal, or environmental events that regulate brain repair (Cramer and Riley, 2008; Varadarajan et al., 2022). Likewise, both neuronal and non-neuronal mechanisms, such as neurogenesis, angiogenesis, astrogliosis and oligodendrogenesis, contribute to the improvement of behavioral deficits in the long-term after ischemic stroke (Font et al., 2010; Hu et al., 2015). Considering the lack of therapeutic drugs that can be administered during the late stage of stroke, it is of great importance to illuminate the mechanisms underlying the complicated repair processes.

As immune cells are essential for both initiating and resolving inflammatory responses, the regulation of brain injury and repair of immune cells are becoming increasingly understood (Seifert and Pennypacker, 2014; Prinz and Priller, 2017; Carrasco et al., 2022). Ischemic damage-induced sterile neuroinflammation, which is caused by recognition by danger/damage-associated molecular patterns (DAMPs) such as ATP, HMGB1 and damaged DNA, and specific pattern-recognition receptors (PRRs), occurs within a few days after stroke onset, during which innate immune cells, including microglia, macrophages, and neutrophils, play a major role. After the acute phase (Days 1–3 post-ischemia), adaptive immunity-associated immune cells such as distinct types of T cells and B cells, have been observed to infiltrate the brain by different means and last for weeks or months, indicating the involvement of these cells in functional recovery (Jayaraj et al., 2019; Iadecola et al., 2020). To date, the contribution of neuroinflammation in the primary injury caused by the initial ischemic event or secondary injury of the brain created by a series of biological and functional changes has long been investigated. The effects of these infiltrated immune cells in the chronic phase of functional restoration are poorly understood.

**FIGURE 1:**
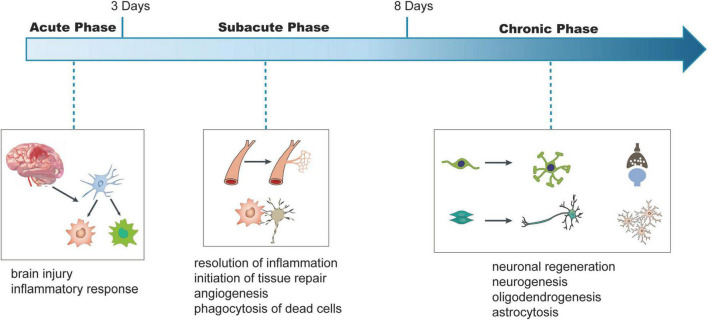
Schematic viewing of time-dependent key injury and repair processes after ischemic stroke. In this review, we refer to Days 1–3 after stroke onset as the acute phase, Days 3–8 being subacute phase, and days exceeding 8 being chronic phase. We illustrate the conceptual changes in each phase. In the acute phase, ischemia in the brain leads to the injury of the brain and inflammatory responses. In the subacute phase, the resolution of inflammatory response and phagocytosis of dead neurons were observed and tissue repair processes, such as angiogenesis, are initiated. During the chronic phase, processes, such as neurogenesis, oligodendrogenesis, and astrocytosis are initiated to regulate neuronal regeneration and functional recovery.

This review discusses the role of infiltrating peripheral immune cells in the functional recovery of ischemic stroke. Besides peripheral immune cells, the brain also harbors some immune cells, which are essential for brain development and function and regulate ischemic stroke (Colonna and Butovsky, 2017; Smolders et al., 2018; Badimon et al., 2020). Many previous studies have thoroughly reviewed the role of brain resident immune cells in ischemic stroke as well as immune cells in the regulation of the acute injury phase (Iadecola and Anrather, 2011; Hu et al., 2015; Jian et al., 2019; Iadecola et al., 2020). Here, we provide focused attention only on the role of peripheral innate and adaptive immune cells in the recovery of neurological function during the subacute (Days 4–8 post-ischemia) and chronic phases (exceeding 9 days post-ischemia) of ischemic stroke (Figure 1), and highlight outstanding questions for future studies.

## Neuronal and non-neuronal mechanisms of functional recovery after ischemic stroke

Ischemic stroke-induced injury can trigger some repair processes, such as neurogenesis, axon sprouting, oligodendrogenesis, angiogenesis, and astrogliosis, the degree of which depends on the severity and topography of the injury. For many decades, findings from human and animal studies have continued to shed light on the molecular, vascular, glial, neuronal, and environmental events that regulate neurological recovery during the weeks after ischemic stroke (Carmichael, 2006; Cramer and Riley, 2008). In the following section, we briefly summarize the main aspects from the neuronal and non-neuronal perspectives (Table 1).

**TABLE 1:** Mechanisms of peripheral immune system in functional recovery after ischemic stroke.

Mechanism		Immune cells or factors	Effects on functional recovery
Neuronal	Neurogenesis	Treg cells (Brifault et al., 2015), MMs (Jones et al., 2018), IL-6 (Alia et al., 2017), IL-17A (Miron et al., 2013), B cells (Chen et al., 2022)	Improvement (Treg cells, B cells, IL-6, IL-17A); Exacerbation (MMs)
	Axon sprouting and Extension	Not reported	
	Synaptic plasticity	Not reported	
	Circuit Reformation	Not reported	
Non-neuronal	Angiogenesis	Neutrophil (Otxoa-de-Amezaga et al., 2019), MMs (Gendron et al., 2002; Chamorro et al., 2012)	Improvement (Neutrophils, MMs)
	Astrogliosis	Treg cells (Qiu et al., 2021)	Improvement (Treg cells)
	Oligodendrogenesis	Treg cells (Martinez and Gordon, 2014), IL-4 (Laterza et al., 2017)	Improvement (Treg cells)
	M1/M2 polarization	IL-4 (Ma et al., 2017), Treg cells (Martinez and Gordon, 2014), MMs (Jin et al., 2006; Lei et al., 2012; Roy Choudhury et al., 2014; Kanazawa et al., 2019; Hatakeyama et al., 2020; Zhu et al., 2021)	Improvement (IL-4, Treg cells, MMs)
	Phagocytosis	MMs (Iwai et al., 2010; Zhang et al., 2010, 2013; Chu et al., 2014; Choudhury and Ding, 2016; Sims and Yew, 2017)	Improvement (MMs)

MMs, myeloid and macrophages; Treg cell, Regulatory T cells; IL-4, interleukin 4; IL-6, interleukin 6; IL-17A, interleukin 17A.

Tremendous efforts have been devoted to characterizing the cellular and molecular mechanisms of neuronal regeneration. Methods to promote neuron regeneration in the brain, such as by blocking inhibitory factors for myelination and promoting axon sprouting, have met with mixed success, albeit some challenges. For example, in many experiments, increased axon sprouting or neuronal survival has been observed after treatment (Barker et al., 2018; Joy et al., 2019; Hu et al., 2020); however, approaches to fully restore circuit function are lacking. In addition, the differences in reactions and effects in animal models and human patients make it difficult for clinical trials (Carmichael, 2006; Carmichael et al., 2017; Varadarajan et al., 2022). These discoveries cannot be ignored as they reveal possible neuronal regeneration mechanisms existing in stroke models weeks and months after acute damage and raise important strategies to intervene in neuronal regeneration and functional recovery, which include neurogenesis, axon sprouting and extension, synapse reformation and stimulation-based refinement of newly formed circuits (Small et al., 2013; Joy and Carmichael, 2021; Shi X. et al., 2021; Varadarajan et al., 2022). To achieve better functional connectivity, methods targeting multifaceted processes are needed.

Besides neuronal injury, ischemic stroke can also result in marked damage to the BBB and neurovascular unit dysfunction, and induce inflammatory responses in glial cells in human and animal models (Jiang et al., 2018; Jayaraj et al., 2019). These changes further lead to events including (1) angiogenesis, which is mediated by endothelial cells; (2) the astrogliosis of astrocytes; (3) oligodendrogenesis, which is regulated by oligodendrocyte progenitor cells (OPCs); and (4) changes in inflammatory phenotypes and phagocytic capacity of innate immune cells (Hatakeyama et al., 2020; Shibahara et al., 2020; Zhu et al., 2021). The capacity of neuronal regeneration and the ultimate recovery of sensorimotor and language deficits or neuropsychiatric sequelae depend on these processes in the long-term.

Angiogenesis is thought to contribute to functional recovery in two ways. First, new blood vessels that are formed after ischemia enhance neurogenesis by facilitating the migration of neural stem/progenitor cells (NSCs/NPCs) toward the infarct region by supplying nutrients, oxygen and soluble factors, promoting the proliferation and differentiation of NSCs/NPCs *via* the expression of extracellular signals and supplying oxygen and growth factors (Grade et al., 2013; Ruan et al., 2015; Lange et al., 2016; Kanazawa et al., 2019). Second, postischemic angiogenesis contributes to axonal outgrowth by vascular endothelial growth factors (VEGFs) and laminin/β1-integrin signaling (Jin et al., 2006; Lei et al., 2012; Hatakeyama et al., 2020). After ischemic injury, some activated astrocytes can transform into reactive astrocytes, which causes astrogliosis or forms glial scars. Although some studies have shown that reactive astrogliosis with compact glial scar formation may impede axonal regeneration and hinder functional recovery process (Zhang et al., 2010; Roy Choudhury et al., 2014), other studies have also demonstrated that reactive astrogliosis can be neuroprotective by generating and releasing factors or proteins such as glial-derived neurotrophic factor (GDNF), VEGF and heterodimeric glycoprotein, clusterin 1 (Choudhury and Ding, 2016; Sims and Yew, 2017). Stroke acutely induces mature oligodendrocyte damage, leading to loss of myelin. During the recovery phase of ischemic stroke, there is a significant increase in the generation of OPCs, and some of them become mature myelinating oligodendrocytes, which are essential for white matter repair and long-term functional recovery after ischemic stroke (Iwai et al., 2010; Zhang et al., 2013). The role of inflammatory phenotypes and phagocytic capacity of innate immune cells in functional recovery are complex and discussed in detail in the peripheral immune cell and functional recovery sections.

## Effects and mechanisms of peripheral immune cells in functional recovery after ischemic stroke

Immediately following ischemic stroke, brain-resident immune cells such as microglia and astrocytes are activated to respond to ischemia injury. Subsequently, peripheral immune cells are activated and recruited to the brain to assist in the immune response (Chu et al., 2014; Jones et al., 2018). In the following days, peripheral immunodepression can occur, with a subsequent increased risk for systemic infections, especially in patients with large strokes (Gendron et al., 2002). The extent of these local and peripheral immune responses to stroke is variable, and this plays an important role in determining patient outcomes and overall functional recovery in the acute and chronic phases after stroke (Chamorro et al., 2012; Hu et al., 2015). In the following part, we summarized the roles of peripheral immune cells in two perspectives: (1) the roles of main immune cells on functional recovery (see section “Peripheral immune cells and functional recovery”); (2) roles of interactions and communications between peripheral immune cells and brain resident cells on functional recovery (see section “Roles of interactions and communications between peripheral immune cells and brain resident cells in functional recovery”).

### Peripheral immune cells and functional recovery

#### Innate immune cells

##### Neutrophils

Neutrophils are traditionally recognized as the first line of innate immune defense against pathogens (Amulic et al., 2012). Similarly, neutrophils are the first responders to infiltrate the brain after ischemic injury as they were observed to attach the brain endothelial cells within a few minutes and peak at 1–3 days (Perez-de-Puig et al., 2015; Ma et al., 2021). After activation, neutrophils produce cytokines to recruit other immune cells, engulf microbes *via* receptor-mediated phagocytosis, and further release granular antimicrobial molecules as well as the formation of neutrophil extracellular traps (NETs) (Amulic et al., 2012). Consistent with their infiltration time, neutrophils mainly function in the acute injury events, such as regulation of BBB integrity and inflammation-mediated brain infarction (Jickling et al., 2015; Cai et al., 2020; Ma et al., 2021).

Recently, one study showed that neutrophils accumulate in the peri-infarct area during all stages of ischemic stroke, and depletion of neutrophils reduces the breakdown of BBB and enhances neovascularization at Day 14 (Kang et al., 2020). Mechanistically, NETs formation promotes subsequent activation of STING-dependent type I IFN production, which potentially induces vascular remodeling to enhance functional recovery (Kang et al., 2020). Notably, a unique immature neutrophil subset, that secretes growth factors to promote axon regeneration in the optic nerve and spinal cord has been identified in a recent report (Sas et al., 2020). Whether this type of neutrophils also enhance axon sprouting in ischemic stroke is unknown.

**FIGURE 2:**
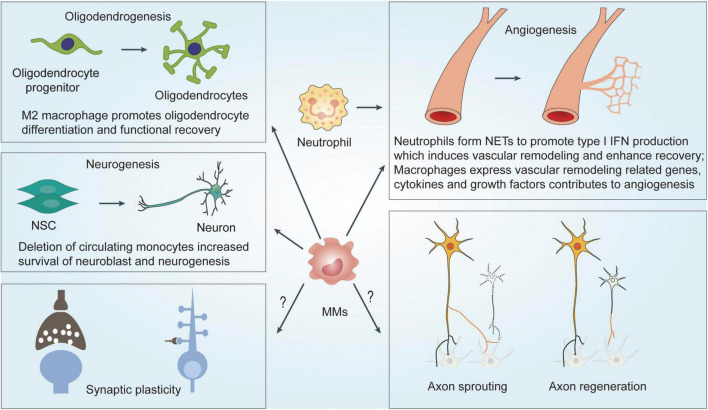
Mechanisms underlying MMs and neutrophils-mediated effects on functional recovery after ischemic stroke. MMs regulates functional recovery by modulating neurogenesis, angiogenesis and oligodendrogenesis. NETs formation of neutrophils induces vascular remodeling and angiogenesis to enhance functional recovery. MMs, Monocytes and macrophage; NSC, neural stem cell; NET, neutrophil extracellular traps; IFN, interferons.

Similar to monocytes and macrophages (MMs), neutrophils can also be derived into proinflammatory N1 and anti-inflammatory N2 subtypes; the N1 type is generally considered neurotoxic and the N2 type is neuroprotective during the acute injury phase of ischemic stroke (Cuartero et al., 2013; Jickling et al., 2015; Wanrooy et al., 2021). Whether type N2 neutrophil-induced resolution of neuroinflammation facilitates functional recovery remains unknown. In addition, studies have shown that microglia mediate phagocytosis of neutrophils, which may help to restore the homeostasis of the brain and retain neuronal function and survivability after stroke (Neumann et al., 2008; Otxoa-de-Amezaga et al., 2019) (Figure 2). The role of different neutrophil subsets in other points of neuroplasticity remains unclear.

##### Monocytes and macrophages

Monocytes and macrophages are mononuclear phagocytes and are derived from macrophage/dendritic cell progenitors in the bone marrow (BM). At least two subtypes of monocytes have been reported in mice: Ly6C*^hi^* classical inflammatory monocytes and Ly6C*^low^* non-classical patrolling monocytes. Ly6C*^low^* monocytes are derived from Ly6C*^hi^* monocytes in the blood or common monocyte progenitor (cMoP) in the BM (Ginhoux and Jung, 2014). During disease progression, they can exit to the circulatory system in a C-C chemokine receptor 2 (CCR2)-dependent manner and enter tissues to give rise to tissue macrophages referred to as monocyte-derived macrophages (MDMs), which express high levels of CD68 (Auffray et al., 2009; Ginhoux and Jung, 2014; Li and Barres, 2018; Han et al., 2020).

###### Monocyte and macrophage responses and functional recovery after ischemic stroke

(1) **Neuroinflammation and functional recovery**

Previous studies showed that the number of monocytes peaks at Day 3 after ischemic stroke, and they differentiate into MDMs (Wattananit et al., 2016; Fang et al., 2018). During ischemic stroke, dying/dead neurons release DAMPs, such as ATP, HMGB1, damaged DNA and peroxiredoxin family proteins, which can be recognized by pattern recognition receptors (PRRs), including Toll-like receptor (TLR)-2 and TLR-4, expressed by some innate immune cells, such as MMs, microglia and neutrophils (Shichita et al., 2012).

**FIGURE 3:**
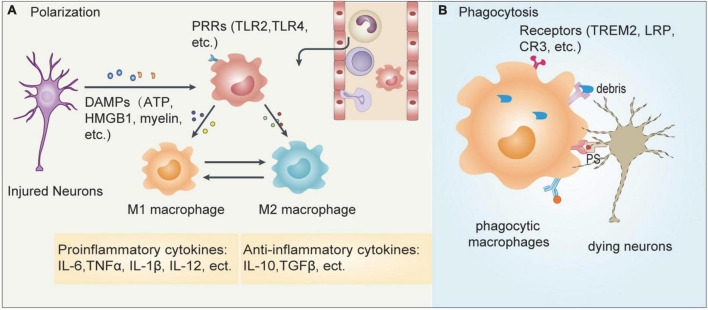
Responses of MMs after ischemic stroke. Cerebral ischemia leads to the release of many inflammatory mediators which contributes to the recruitment of peripheral immune cells into the infarct brain. **(A)** The dying/dead neurons release damage-associated molecular patterns (DAMPs) such as ATP, HMGB1, myelin, etc., which can be recognized by pattern recognition receptors (PRRs) such as TLR2 and TLR4 expressed on MMs. The activated macrophages can then polarize into pro-inflammatory M1 or anti-inflammatory M2 cells. **(B)** In addition, macrophages can also recognize phosphatidylserine (PS) expressed on apoptotic neurons or myelin debris released from injured neurons, which is mediated by some specific receptors including TREM2, lipoprotein receptor-related protein LRP and complement receptor CR3, leading to phagocytosis. These two events coordinately regulate the outcome of ischemic stroke.

Many studies showed that activated MMs can polarize into distinct subtypes, including the well-known M1 and M2 subpopulations (Figure 3A). The M1 subtype secretes proinflammatory cytokines, such as tumor necrosis factor alpha (TNF-α), interleukin (IL)-1β, IL-12, and IL-6, and can be distinguished by cell surface markers CD16 and CD32. The M2 phenotype produces TGF-β, IL-4, IL-10, and IL-13, and expresses CD206 and Arg1. The activation of MM subpopulations and other innate immune cells leads to neuroinflammation (Hu et al., 2012; Wattananit et al., 2016; Fang et al., 2018), the role of which has been well characterized in the acute phase of ischemic brain injury (Jayaraj et al., 2019; Qiu et al., 2021). During the subsequent 2 weeks, MMs gradually shift from the proinflammatory M1 phenotype to the alternatively activated M2 phenotype, facilitating the resolution of inflammation (Wattananit et al., 2016; Fang et al., 2018). However, such definition may not fully illustrate all different activation scenarios. As many reports proposed that there are some other subtypes between M1 and M2, such as M2a, M2b, and M2c (Martinez and Gordon, 2014). With the development of single-cell sequencing technique, the different phenotypes of MMs subpopulations during different stages of ischemic stroke needs further investigation.

Early studies showed that strategies to switch MMs from the proinflammatory phenotype to the anti-inflammatory phenotype promote long-term functional recovery (Brifault et al., 2015; Zheng et al., 2019; Hou et al., 2021; Liu et al., 2021). For example, delayed pituitary adenylate cyclase-activating polypeptide delivery after cerebral ischemia enhances functional recovery by inducing the polarization of MMs toward M2 phenotype (Brifault et al., 2015). Poststroke treatment with TGFβ-activated kinase 1 (TAK1)-specific inhibitor 5Z-7-oxozeaenol (OZ) causes a phenotypic shift in microglia/macrophages toward an inflammation-resolving state, which effectively promotes the integrity of both gray matter and white matter and long-term neurological recovery (Jickling et al., 2015). In addition, MMs can also express receptors, such as Mannose receptors and macrophage scavenger receptor-1 Msr1, to facilitate the clearance of DAMPs, and contributes to inflammation resolution and brain repair (Giraldi-Guimaraes et al., 2012; Shichita et al., 2017). Moreover, one study showed that modulation of monocytes by supplementation with ursolic acid prevents monocyte dysfunction in diabetic mice and protects mice against atherosclerosis and loss of renal function (Ullevig et al., 2014). Given that many metabolites are altered after ischemic stroke (Wang X. et al., 2020), whether modulation of metabolic of MMs can alter their cytokine profile and affect functional recovery deserve further investigation. For microglia, the polarization from the M1 to the M2 phenotype promotes functional recovery by facilitating neurovascular remodeling, neurogenesis, white matter integrity, and neuroplasticity (Hu et al., 2015; Lyu et al., 2021). Whether the phenotypic switch from M1 to M2 of MMs also promotes brain repair as microglia do still needs further investigation.

(2) **Phagocytosis and functional recovery**

Phagocytosis of dead/dying neurons or myelin debris is another important response of MMs to modulate brain repair (Figure 3B). When ischemic stroke occurs, several substances, including ATPs, sphingosine-1-phosphate, and chemokines, are released by injured neurons that act as “find me” signals, which attract neighboring phagocytes to migrate to the injury site. Phagocytes can sense and recognize specific “eat me” signals, such as phospholipid phosphatidylserine, calreticulin, or the complement components C1q and C3b, which are present on the dead or dying cells *via* specific receptors, including TREM2, lipoprotein receptor-related protein LRP and complement receptor CR3 to mediate phagocytosis (Jia et al., 2021; Chen et al., 2022). On one hand, the phagocytosis of dead neurons or myelin debris can help to prevent the release of cytotoxic intracellular contents, which act as DAMPs to promote inflammation and lead to secondary damage, and on the other hand, the phagocytosis of dead/dying neurons modulates tissue reconstruction and neuronal network reorganization to promote functional recovery (Jia et al., 2021; Lyu et al., 2021).

At present, compared with microglia, whose regulatory mechanisms and cellular pathways of phagocytosis, and their resulting effects in neural regeneration are well studied, the detailed mechanism of action of peripheral MMs is less understood. RNA-seq results showed that a large number of genes related to chemotaxis, the recognition of dead cells, engulfment, and the processing of phagosomes are upregulated in brain-infiltrating macrophages after ischemic stroke, and they promote the phagocytosis of dying neurons, which facilitates inflammation resolution, and finally the recovery of neurological functions (Zhang W. et al., 2019). Factors such as CD36, TREM2, Kv1.3, and STAT6/Arg1 contribute to the MM-mediated phagocytosis of dying neurons and enhance functional recovery (Woo et al., 2016; Cai et al., 2019; Gao et al., 2019; Hu et al., 2021). Moreover, the microvascular pericytes colocalize with macrophages within the infarct area and potentiate the clearance activity of recruited macrophages toward myelin debris, which subsequently stimulates oligodendrogenesis and remyelination after ischemia (Shibahara et al., 2020).

In conclusion, during ischemic stroke, MM-mediated inflammation and phagocytosis are dynamically altered. The inflammatory and phagocytic response of MMs can be different in the acute, subacute, and chronic phase by time, and in the ischemic core or the peri-infarct region by location (Chen et al., 2022). In addition, the M1 and M2 phenotype have different phagocytosis capacities, although they both express phagocytic receptors. The M2 phenotype is more efficient at clearing dead cells than the M1 phenotype (Kapellos et al., 2016). Currently, it remains unknown, how the inflammatory phenotype and phagocytic capacity of MMs are cross-regulated, and even less is known about the detailed molecular mechanisms and their effects on functional recovery after ischemic stroke. Therefore, future studies need to consider the inflammatory phenotype and the phagocytosis of MMs in the context of time and location to optimize treatment for improving functional recovery.

###### Diverse ways of monocytes and macrophages in regulating functional recovery after ischemic stroke

(1) **Neurogenesis and angiogenesis**

Accumulating evidence suggests that microglia can regulate functional outcomes after ischemic stroke *via* diverse mechanisms including neurogenesis, angiogenesis, oligodendrogenesis, and neuroplasticity (Ma et al., 2017; Jia et al., 2021; Lyu et al., 2021). Similarly, MMs also seem to regulate neurological function from these aspects besides some differences. Depletion of circulating monocytes by using CCR2 antibody MC-21 early after stroke enhances neurogenesis in the subventricular zone (SVZ) as well as migration of neuroblasts toward the damaged striatum (Laterza et al., 2017). Pro-reparative monocytes facilitate angiogenesis and improve behavioral performance after ischemic stroke (Pedragosa et al., 2020). Further transcriptome analysis of infiltrated MMs in a mouse model of permanent focal cerebral ischemia indicated that neurovascular remodeling associated genes, such as Wnt, epidermal growth factor receptor (EGFR), and Notch signaling-related genes involved in angiogenesis and neurogenesis, and cytokines, growth factors that contribute to angiogenesis and neuroplasticity, are overexpressed. Myeloid cell-specific deletion of PPARc reduced poststroke angiogenesis and neurogenesis, and exacerbated neurological deficits (Wang R. et al., 2020). These data suggest that MMs modulate functional recovery *via* neurogenesis and angiogenesis, although the detailed molecular mechanism remains unclear.

(2) **Neuroplasticity**

Neuroplasticity enables the restoration of neural networks and rewiring of functional connections after ischemic stroke (Alia et al., 2017). In recent years, increasing evidence demonstrated the involvement of microglia in the regulation of neuroplasticity (Hu et al., 2015; Ma et al., 2017; Lyu et al., 2021). For MMs, a close anatomical association between activated macrophages and sprouting dopaminergic axons was observed, and macrophages at the wound edges highly express neurotrophic factors that support axon sprouting (Batchelor et al., 2002). Oligodendrogenesis, mediated *via* OPC proliferation and differentiation, promotes remyelination, which influences the integrity of white matter and affects signal transmission efficiency and neuroplasticity after ischemic stroke (Miron et al., 2013). An *in vivo* study showed that depletion of M2 cells including M2 macrophages inhibits oligodendrocyte differentiation (Miron et al., 2013). Consistent with this finding, OPC differentiation can be stimulated by macrophage-conditioned medium with myelin debris *in vitro* (Shibahara et al., 2020). In addition, proinflammatory cytokines, such as IL-6 and IL-1β, play an important role in synaptogenesis and long-term potentiation during development (Katsuki et al., 1990; Faust and Schafer, 2021), although the role of pro-inflammatory or anti-inflammatory cytokines in the modulation of synaptic plasticity and thus potentially brain plasticity is far less clear. Illuminating cellular mechanisms of MMs in the functional recovery of ischemic stroke as well as the detailed molecular mechanisms may lead to novel therapeutic strategies.

#### Adaptive immune cells

##### T cells

T cells are key players in cellular adaptive immunity, which functions in a variety of neurological diseases (Prinz and Priller, 2017; Carrasco et al., 2022). In response to the upregulation of adhesion molecules on endothelial cells and the exposed CNS antigens from the injured brain, T cells can be activated and then invade the brain. Three different routes of T lymphocyte migration exist: (1) the BBB pathway, (2) the meninges and choroid plexus (ChP) infiltration routes, and (3) the ChP stroma pathway (Llovera et al., 2017; Benakis et al., 2018). According to the previous reports, the day of T-cells infiltration peak varied, with some studies showing an infiltration peak within 24 h, some around Day 3 to Day 7, and some in the chronic phase, which may result from different stroke models and testing methods (Gronberg et al., 2013; Stubbe et al., 2013; Chu et al., 2014).

The role of different T-cell subsets in ischemic stroke in the acute damage phase remains controversial (Zhang et al., 2021). Blocking CD8^+^ T-cell expansion and activation through the administration of IL-2 or IL-15 neutralizing antibody, or depletion of CD8^+^ T cells with anti-CD8α antibody could significantly reduce brain infarct volume and attenuate the associated behavioral deficits in two ischemia models that rely on the production of perforin (Mracsko et al., 2014; Lee et al., 2018; Zhou et al., 2019). The mode of CD4^+^ T-cell differentiation in response to brain injury ultimately determines stroke outcome. IFN-γ released from Th1 cells appears to either worsen outcomes (Lambertsen et al., 2004; Yilmaz et al., 2006) or have an effect on brain infarct volume (Shichita et al., 2009). Loss of IL-4 or neutralization of IL-4, the main cytokines released from Th2 cells, exert a neuroprotective effect (Korhonen et al., 2015; Xiong et al., 2015; Zhang et al., 2018). Interestingly, the infiltrating IL-17 producing cells in the brain are not CD4^+^ Th cells, but γδ T lymphocytes (Swardfager et al., 2013). The balance in the peripheral Treg/Th17 cells ratio is altered after stroke which in turn modifies stroke pathophysiology. For example, conditional knockout of ACC1 (acetyl coenzyme A carboxylase 1), a key enzyme involved in *de novo* fatty acid synthesis, profoundly alleviates ischemic brain injury by preserving the balance of Treg/Th17 cells (Wang et al., 2019), indicating the involvement of cell metabolism in stroke pathogenesis. Inconsistent with the long existence of T cells in the brain after ischemic stroke, the role of different T-cell subsets, especially Treg cells in the chronic phase of functional recovery is being exposed.

**FIGURE 4:**
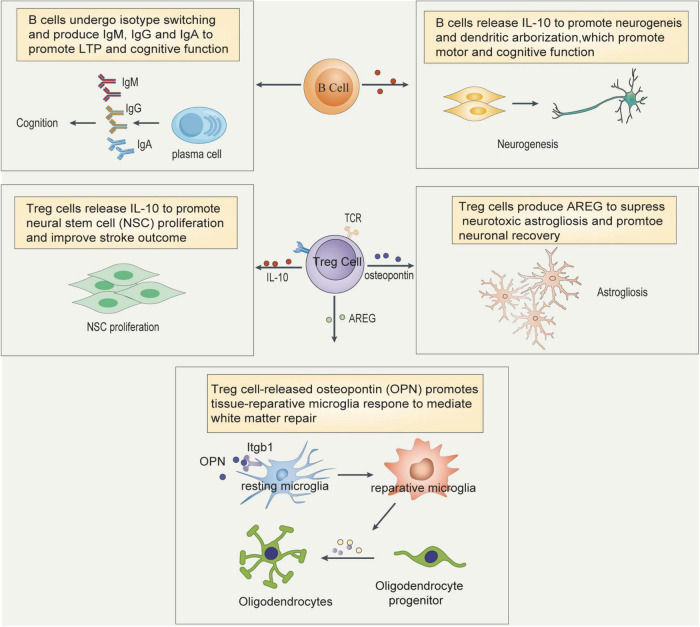
Mechanisms underlying Treg cell and B cell-mediated impacts on functional recovery after ischemic stroke. Treg cells promotes functional recovery after ischemic stroke by the following means: Treg cells release IL-10 to directly promote neural stem cell proliferation or restrict proinflammatory cytokine production; Treg cells release AREG to inhibit astrogliosis and facilitate neuronal recovery; Treg cell-derived osteopontin act through integrin receptors on microglia to enhance microglial reparative activity, as both anti-inflammatory cytokines and genes encoding proteins involved in brain repair are upregulated, consequently facilitating oligodendrocyte regeneration and white matter integrity. B cells release IL-10 to promote neurogenesis and dendritic arborization, which promote motor and cognitive function, and in addition, B cells also undergo isotype switching and produce IgM, IgG and IgA to promote cognitive function. TCR, T cell receptor; ILRs, interleukin receptors; AREG, amphiregulin; NSC, neural stem cell.

Treg cells are essential for maintaining immune homeostasis by suppressing conventional T cell activation and function (Zhu et al., 2010). Accumulating evidence indicates that Treg cells migrate into the brain in the chronic phase of ischemic stroke and last for several months, during which Treg cells exhibit multifaceted roles to promote functional recovery (Wang et al., 2015; Ito et al., 2019; Shi L. et al., 2021). First, Treg cells release AREG to suppress astrogliosis and increase brain recovery (Ito et al., 2019). Second, Treg cell-derived osteopontin can act through integrin receptors on microglia to enhance microglial reparative activity, consequently facilitating oligodendrocyte regeneration and white matter integrity (Shi L. et al., 2021). Third, NSC proliferation in the SVZ of normal and ischemic brain can also be enhanced by activated Treg cells *via* IL-10 (Wang et al., 2015) (Figure 4). The role of Treg cells on neuroplasticity especially axon sprouting and synaptic function, and other T cell subsets in the functional recovery remain to be established.

Similar to CD4^+^ T cells, CD8^+^ T cells also persist in the injured brain for weeks. One study showed that mice with higher ipsilesional CD8^+^ T cells at Day 30 exhibited worse functional recovery. The Depletion of CD8^+^ T cells beginning 10 days post-tMCAO improved motor recovery (Selvaraj et al., 2021). Notably, one recent report discovered a new CD8+ T regulatory-like cells (CD8^+^CD122^+^CD49d*^lo^*), which could reprogram to upregulate leukemia inhibitory factor (LIF) receptor, epidermal growth factor–like transforming growth factor (ETGF), and interleukin 10 (IL-10) to exert neuroprotection and promoted long-term neurological recovery (Cai et al., 2022). In contrast, traumatic brain injury activates CD8^+^ T cells, which causes long-term neurological impairment in mice (Daglas et al., 2019). The role of CD8^+^ T cells in neuroplasticity is worth investigation.

##### B cells

B cells were originally identified through studies searching for the cellular source of antibodies. As the key player in humoral immunity, B cells contribute to immunity through antigen presentation, antibody production and cytokine secretion (Jain and Yong, 2021). The role of B cells in the acute phase of ischemic stroke is inconclusive. Some studies found no effect on infarct and stroke outcome (Kleinschnitz et al., 2010; Schuhmann et al., 2017). However, others observed a beneficial role of B cells (Ren et al., 2011; Offner and Hurn, 2012).

Given that the adaptive immune response specific for CNS antigens develops later than the innate immune response, the role of B cells in the subacute and chronic phase of ischemic stroke attracts has attracted the attention of some researchers’. Ischemic injury induces significant bilateral B-cell diapedesis into remote brain regions to regulate motor and cognitive functions by supporting neuronal viability and dendritic arborization (Ortega et al., 2020). In a distal middle cerebral artery occlusion (dMCAO) model, B cells were found to infiltrate the infarct region and secrete IgA and IgG in the chronic phase after stroke, which may directly impact cognition after stroke (Doyle et al., 2015; Figure 4). Notably, multiple sclerosis studies have raised the notion that B cells contribute to CNS pathology independent of antibody production (Li et al., 2018). Consistent with this finding, B cells have been reported to produce several neurotrophins including brain-derived neurotrophic factor (BDNF) and nerve growth factor (NGF) (Edling et al., 2004; Fauchais et al., 2008). Whether B cells regulates other events in neurological recovery in an antibody-production-independent manner is a field worth future study.

##### Natural killer cells

Natural killer (NK) cells are a type of cytotoxic lymphocyte that play an important role in innate immunity (Abel et al., 2018; Chen et al., 2019). NK cells were reported to accumulate in the brain during the acute injury phase. Kinetic experiments showed that NK cells accumulated as early as 3 hours, peaking at Day 3 after tMCAO (Gan et al., 2014). In human ischemic brain, the infiltrations of NK cells peaked at Days 2–5 (Zhang et al., 2014). Consistent with their infiltration time, NK cells contribute to brain injury in various ways: they induce the necrosis of neural cells *via* IFN-γ (Chen et al., 2019); damage the BBB in response to interferon-inducible protein-10 (IP-10) (Zhang et al., 2014); mediate cytolytic killing of ischemic neurons *via* perforin (Gan et al., 2014); act in cooperation with monocytes and platelets to propagate thrombosis and activate the complement system (Eltzschig and Eckle, 2011). At present, the function of NK cells on neurofunctional recovery is poorly understood.

Stroke-induced systemic immunosuppression was first described in the 1970s and is characterized by severe lymphopenia in the peripheral blood, thymus, and spleen, which affects the mortality and functional recovery of patients with ischemic stroke (Offner et al., 2006; Klehmet et al., 2009; Wong, 2019). Currently, stroke-induced immunosuppression is considered both detrimental and beneficial to the human body. Stroke-induced immunosuppression is deleterious, as it increases the incidence of poststroke infections, especially pneumonia and urinary tract infection (Iadecola and Anrather, 2011; Chamorro et al., 2012). Several studies have provided a mechanistic basis for this phenomenon and demonstrated that T lymphocytes and iNKT cells play critical roles in regulating poststroke induced immunosuppression as well as subsequent infections (Prass et al., 2003; Wong et al., 2011; Roth et al., 2021). The spleen is reported to exacerbate brain injury and contributes to long-term neurodegenerations (Ajmo et al., 2008; Pennypacker and Offner, 2015). After brain ischemia, spleen atrophy and peripheral NK Cell cells were observed. NK cells display remarkably distinct temporal and transcriptome profiles in the brain and spleen. Catecholaminergic and hypothalamic-pituitary-adrenal (HPA) axis innervations suppress peripheral NK cells after ischemic stroke. Correspondingly, modulation of neurogenic pathways preserves NK cell function and improves host immune defense against poststroke infections (Liu et al., 2017).

### Roles of interactions and communications between peripheral immune cells and brain resident cells in functional recovery

Accumulating evidence shows that the infiltrated peripheral immune cells interact with brain resident cells in complex ways, that may be either detrimental or supportive for brain injury and repair. After ischemic stroke, the bidirectional neuroimmune interactions and communications of infiltrated peripheral immune cells and brain resident cell allow these cell populations to influence each other in multiple ways, including releasing factors to affect each other and direct cell–cell interactions (Kamel and Iadecola, 2012; Alia et al., 2021; Muhammad et al., 2021).

#### Communications-mediated by cytokines or other factors and functional recovery

After brain injury, various DAMPs, such as ATP, HMGB1, damaged DNA and peroxiredoxin family proteins and brain-derived antigens, can be released after ischemic insult into the periphery, which activates the peripheral immune cells and leads to further infiltration into the brain owing to a compromised BBB (Javidi and Magnus, 2019; Stanzione et al., 2022). These infiltrated activated immune cells or autoreactive adaptive immune cells can then polarize or differentiate into different populations, such as M1/M2 subtypes and different Th subsets, which further interact with other brain resident cells to modulate brain repair. For example, brain infiltrating Treg cells can on one hand release AREG to suppress astrogliosis and increase brain recovery (Ito et al., 2019), and, on the other hand, produce osteopontin to activate integrin receptors on microglia and enhance microglial reparative activity to facilitate oligodendrocyte regeneration and white matter integrity (Shi L. et al., 2021). In addition, during autoimmune disease or tumor progression, the interaction between macrophage and T cells was observed and their interaction affect the progression of the disease (Han et al., 2012; Cess and Finley, 2020). On one hand, cytokines or chemokines produced by T cells can lead to recruitment and activation of macrophages, which further produce additional inflammatory mediators and become effector cells in pathogenesis (Yoon and Jun, 1999); On the other hand, macrophages can present antigen to T cells, which induce their activation and differentiation to affect disease progression (Underhill et al., 1999). The role of macrophage and T cell interaction in the functional recovery of ischemic stroke is still unclear.

After activation, both adaptive and innate immune cells secrete cytokines, which play multiple and vital roles in neurological disease progression (Kerschensteiner et al., 2010; Cui and Wan, 2019). Some studies have supplemented certain cytokines during ischemic stroke to study their direct effects (Table 1).

Interleukin-4 (IL-4) is an anti-inflammatory cytokine produced by a variety of immune cells, including Th2 cells, mast cells, eosinophils and basophils (Gadani et al., 2012), and can drive macrophages into M2 phenotype in the presence of IL-13 (Murray, 2017). Loss of IL-4 promotes the M1 phenotype in microglia/macrophages after ischemic stroke and exacerbates long-term sensorimotor dysfunction as well as cognitive deficits after ischemia, while infusion of IL-4 into the cerebroventricular after ischemic stroke improves neurological functions (Liu et al., 2016). In addition to promoting M2 phenotype polarization, intranasal delivery of IL-4 nanoparticles poststroke also improves white matter integrity by acting directly on OPCs to enhance oligodendrocyte differentiation mediated by the PPARγ axis, which reduces long-term sensorimotor and cognitive deficits (Zhang Q. et al., 2019).

Tumor necrosis factor alpha and IL-33 have also been found to confer a protective effect on regulation of white matter integrity. TNFα directly protected OPCs and oligodendrocytes against oxygen and glucose deprivation (OGD)-induced cell death, but did not affluence on OPC differentiation, which is mediated by the EGFR and the downstream transcription factor STAT3 on oligodendrocyte lineage cells (Dai et al., 2020). IL-33 also protected oligodendrocytes and OPCs against ischemic injury but in a microglia/macrophage-dependent manner by promoting a beneficial response *via* the ST2-ATAT6 axis (Xie et al., 2021).

In contrast to IL-4, IL-6 is a proinflammatory cytokine secreted by some innate immune cells. Mice treated with recombinant IL-6 exhibited significantly increased proliferation and neural differentiation of NPCs in the ipsilateral SVZ, as well as functional recovery. However, IL-6 neutralizing antibody confer the opposite effects (Meng et al., 2015). Injection of IL-6 in pregnant mice or developing embryos increases glutamatergic synapse density and overall hyperconnectivity in the offspring (Mirabella et al., 2021); thus future studies should clarify the role of IL-6 in poststroke synapse plasticity.

Leukemia inhibitory factor is a multi-functional cytokine belongs to the IL-6 cytokine family. LIF modulates post-stroke responses and reduces infarct volume (Davis et al., 2017; Davis and Pennypacker, 2018). In addition, LIF also preserves white matter injury and improves functional outcomes when administered to rats subjected to pMCAO. Mechanistically, LIF reduces superoxide dismutase activity through increasing peroxiredoxin 4 (Prdx4) transcripts *via* the Akt signaling (Rowe et al., 2014). Likewise, IL-17A, another proinflammatory cytokine, also enhances the survival and neuronal differentiation of NPCs and subsequent synaptogenesis, which modestly ameliorates functional deficits after stroke (Lin et al., 2016). The role of other cytokines and chemokines in chronic functional recovery still needs further investigation.

Besides the influence of cytokines-released from peripheral innate and adaptive immune cells on brain resident cells, brain resident cells can also regulate peripheral immune cell function through neuroinflammation. After ischemia-induced cell death, activation of microglia and astrocytes were observed and neuroinflammation is initiated (Cekanaviciute and Buckwalter, 2016; Xu et al., 2020). The released cytokines and chemokines can further mediate peripheral immune cell infiltration and modulate their differentiation, which further affects functional recovery (da Fonseca et al., 2014; Dudvarski Stankovic et al., 2016).

##### Direct cell–cell interactions and functional recovery

Peripheral immune cells and brain-resident cells can directly interact with each other and affect neurological function recovery. For example, infiltrating peripheral immune cells are able to interact with brain resident cells, and resolve inflammation and promote functional recovery *via* phagocytosis. *In vitro* studies showed that in organotypic brain slices, externally invading polymorphonuclear neutrophils massively enhanced ischemic neurotoxicity, and microglia exerted protection through the rapid engulfment of apoptotic and motile neutrophils within brain slices. Consistently, in an *in vivo* ischemic stroke model, neutrophils reach the perivascular spaces of brain vessels, and reactive microglia can interact and engulf neutrophils at the periphery of the ischemic lesion to help restore the homeostasis of the brain (Neumann et al., 2008; Otxoa-de-Amezaga et al., 2019). Moreover, pericytes can colocalize with macrophages within the infarct area and potentiate the clearance of debris by recruited macrophages, which subsequently stimulates remyelination after ischemic stroke (Shibahara et al., 2020).

These important and intricate communications and interactions indicate the complexity of therapeutic strategies for clinical application. For example, in the acute phase, some immune cells can produce cytokines and growth factors, which serve as important resources for neuronal sprouting, neurogenesis, angiogenesis, and matrix reorganization (Alia et al., 2021; Varadarajan et al., 2022). If the production of these cytokines or growth factors is blocked, acute brain injury may be reduced, but brain repair may be hindered in the long term hinder. Therefore, attempts to treat stroke patients by modifying these interactions or communications must be made with caution. Future studies should test the proper concentration of the inhibitors or neutralization antibodies, the time to administer these blockers, and specific type of cytokines or growth factors in order to drive the development of immunotherapies of ischemic stroke.

## Discussion

In summary, this review provides a systematic summary of the important role of the innate and adaptive immune systems in regulating brain repair after ischemic stroke and raises some currently unresolved questions. Monocytes and macrophages actively participate in neurogenesis, angiogenesis, and oligodendrogenesis after the acute phase of cerebral ischemia, during which their polarization and phagocytotic function are engaged. Neutrophils can impair revascularization and vascular remodeling by releasing extracellular traps. In addition, evidence for the critical role of Treg cells in facilitating functional recovery has accumulated. B cells may regulate neurological function in an antibody-dependent manner. More importantly, these cells can interact and communicate with each other and the brain resident cells in a very complex way to influence ischemic injury severity and functional recovery. Thus, future clinical trials must consider these stage-specific effects and the complex neuroimmune interactions.

From the reviews above, we could easily realize that there is still a long way to go to understand the regulation of immune cells in neurological recovery in depth, as so many questions remain unknown. For example: (1) What are the effects of the innate and adaptive immune cells on neuroplasticity after ischemic stroke, especially axon sprouting and synaptic plasticity? (2) What are the impacts of the adaptive immune cells on neuropsychiatric consequences of stroke? (3) Is the response of the infiltrating immune cells distinct in different regions of the brain after ischemic stroke? The development of single-cell sequencing technologies may help resolve this question. (4) How the immune cells, especially macrophages, balance their survival, inflammation and phagocytotic function during ischemic stroke? Currently, the progress of metabolomics and chromatin-related technologies such as ATAC-seq, chromatin conformation capture 3C, Hi-C may bring some new prospective. (5) In the microscale or molecular level, how the immune cells interact with neurons or other brain-resident cells, to transmit signals and affect functional prognosis? With the improvement of the resolution of microscopy, to see the interaction between cells at the molecular level may not be impossible. Resolving these questions would move forward the immunity-based translational research focusing on recovery-specific therapy.

## Author contributions

ZZ was involved in the writing, reading literature, design of the figures. ML and XZ contributed to the literature search and editing of the manuscript. YC was involved in the overall supervision of the review and editing of the manuscript. All authors approved the submitted version.
